# Antidepressant-Like Effect of an Immediate-Release Formulation of *Mallotus oppositifolius* in Mice

**DOI:** 10.1155/sci5/7695732

**Published:** 2025-11-27

**Authors:** Zakaria Abdullah Ibrahim, Ofosua Adi-Dako, Kevin Kofi Adutwum-Ofosu, Patrick Amoateng, Frimpong Appiah, Awo Efua Koomson, Donatus Wewura Adongo, Awo Afi Kwapong, Kennedy Kwami Edem Kukuia

**Affiliations:** ^1^Department of Medical Pharmacology, University of Ghana Medical School, College of Health Sciences, University of Ghana, Accra, Ghana; ^2^Department of Pharmaceutics and Microbiology, School of Pharmacy, College of Health Sciences, University of Ghana, Accra, Ghana; ^3^Department of Anatomy, University of Ghana Medical School, College of Health Sciences, University of Ghana, Accra, Ghana; ^4^Department of Pharmacology and Toxicology, School of Pharmacy, College of Health Sciences, University of Ghana, Accra, Ghana; ^5^Department of Community Health and Medicine, School of Food and Health Sciences, Anglican University College of Technology, Nkoranza, Ghana; ^6^Department of Pharmacology, School of Pharmacy, University of Health and Allied Sciences, Ho, Ghana

**Keywords:** brain-derived neurotrophic factor, chronic unpredictable mild stress, forced swim test, immediate-release formulation, *Mallotus oppositifolius*, tail suspension test

## Abstract

**Background:**

Antidepressant-like effects of the crude extract of *Mallotus oppositifolius* (MOE) have been previously demonstrated. However, to overcome the limitations of crude extracts as medicines, we produced an immediate-release formulation of MOE (MOE-IR) and tested its efficacy.

**Methods:**

Using the wet granulation method, MOE was formulated into immediate-release dosage forms (MOE-IR F_1_ and F_2_, 10, 30, 100 mg) and flow properties were assessed with bulk density, tapped density, Carr's index, Hausner's ratio, and the angle of repose. *In vitro* dissolution and antioxidant studies were conducted. Murine unpredictable chronic mild stress and sucrose preference tests (UCMS-SPTs) assessed the antidepressant-like effect. Except for the vehicle nonstressed (VEH-NS) group, mice were subjected to the UCMS for 7 weeks while receiving normal saline (VEH-S, 10 mL/kg; p.o.), MOE-IR (10, 30, and 100 mg/kg; p.o.), or fluoxetine (FLX 20 mg/kg; p.o.) daily for 5 weeks. The weight of mice and sucrose consumption (test for anhedonia) were monitored, after which forced swim test (FST), tail suspension test (TST), and open field test (OFT) were conducted following treatment termination. Plasma corticosterone concentration was assessed using ELISA, and brains were harvested for Golgi-Cox staining.

**Results:**

MOE-IR F_1_ (10 mg) exhibited the most suitable formulation properties, and the highest release profile in all media, hence, was selected for the proof-of-concept antidepressant study and referred to as MOE-IR. MOE-IR and crude extract demonstrated *in vitro* antioxidant activity in the DPPH test. MOE-IR just as FLX reversed the stress-induced weight loss, anhedonia as well as decreased immobility time in the FST and TST without affecting locomotor activity. MOE-IR decreased the plasma corticosterone concentration and increased the dentate gyrus (DG) dendritic spine density.

**Conclusion:**

Collectively, MOE-IR demonstrated antidepressant-like that may be associated with antioxidant effects, decreased plasma corticosterone levels, and increased DG dendritic spine density.

## 1. Introduction

Major depressive disorder is a highly prevalent chronic, recurrent, and disabling psychiatric disorder [1]. It is considered the second leading cause of disability globally and affects an estimated 264 million people [2]. Although several antidepressants are available, these drugs have a success rate of about 60% [3, 4]. Aside full remission of depressive symptoms being elusive, many patients experience unacceptable delays in clinical response to antidepressants [1]. In addition, conventional antidepressants are associated with relapse, intolerable side effects, and negative drug interactions [5]. For these reasons, novel therapeutic strategies and antidepressant drugs are needed to provide more effective relief to patients with major depressive disorder. As a result of their positive effects on human health and well-being, herbal medicines are currently being adopted and utilized as acceptable alternatives where there are limitations to optimizing treatment in conventional therapy [6, 7]. There are ample references to psycho-neuropharmacological effects of herbal medicines [8]. Several phytochemicals in these herbal medicines demonstrate promising effects against depressive symptoms influencing neurotransmitter release similar to established antidepressants [9]. *Mallotus oppositifolius*, an open shrub belonging to the family Euphorbiaceae and distributed widely in Africa across many parts of Nigeria, Angola, Ghana, Cameroon, Senegal, Ethiopia, and Madagascar [10], is one of such medicinal plants. The leaves are reported to possess analgesic, anti-inflammatory, hemostatic, and anthelmintic agents, while the root is known to exhibit bactericidal effects [11–13]. Moreover, previous studies by our laboratory showed that the hydroethanolic crude extract has an anticonvulsant effect, mediated through GABAergic neurotransmission [13], suggesting neuroactive effects. In addition, the extract exhibited a faster antidepressant-like effect in mice when compared to fluoxetine (FLX) and imipramine [14, 15]. The mechanisms of the antidepressant-like effect have been shown to involve an interplay of glycine/NMDA receptor complex and monoaminergic systems [14, 15]. Kwofie et al. [16] established that *M. oppositifolius* exerted its antidepressant action by reversing UCMS induced reduction in serotonin (5_HT_) levels in the prefrontal cortex (PFC) and hippocampus of male ICR mice, which are the main brain areas controlling behavior and cognition as well as the modulation of gut microbiota. Pathogenesis of depression is usually associated with impaired plasticity of hippocampal neurons [17]. The role of inflammation in stress-induced depression has gained traction in recent times, wherein chronic stress can lead to neuroinflammation [18, 19]. Investigations into other herbal formulations that demonstrate anti-inflammatory properties have shown reductions in depressive symptoms under UCMS conditions, hinting at the possibility that *M. oppositifolius* could possess similar traits due to its reported bioactive compounds [20] with reported anti-inflammatory and antioxidant potential [21]. This lays credence to *M. oppositifolius* as a putative alternative to conventional antidepressants in depression management.

Despite their proven efficacy, herbal medicines in the crude extract form have been widely criticized due to a lack of standardization and poor apparent quality. There are limitations in bioavailability due to reduced absorption or extensive first pass metabolism in some cases. Also, crude herbal extracts have poor compressible and very hygroscopic powders with poor powder flowability, which makes their use as medicine in that form unreliable [22]. This has necessitated the formulation of herbal medicines into acceptable dosage forms [23]. The advantages include attaining optimal therapeutic response to a drug incorporated in the formulation and ensuring reproducibility in product quality for large-scale manufacture [24, 25]. Also, formulation into solid dosage forms enhances aqueous solubility and stability, optimizes bioavailability, and ensures dosing accuracy, safety, and efficacy of the drug. This is crucial for patient convenience, compliance, and acceptability [26]. Therefore, the formulation of the *M. oppositifolius* extract into suitable dosage forms can circumvent the current drawbacks of crude extracts of herbal medicines and yield an even better therapeutic response in the treatment of depression. Moreover, fast acting immediate-release formulations will be of immense benefit in curtailing significant lag times with the onset of action with current therapy [27]. Thus, the study aimed at producing immediate-release capsule dosage forms of *M. oppositifolius* with the best flow properties using the wet granulation method as well as investigating drug release properties. The dosage form with the best release profile was selected for proof-of-concept study in mice for the management of depression.

## 2. Materials and Methods

### 2.1. Chemicals and Reagents

Microcrystalline cellulose (MCC), lactose, and sodium carboxymethyl cellulose were obtained from Anhui Sunhere Pharmaceutical Excipients Co. Ltd, Huainan. Potassium dihydrogen phosphate (KH_2_PO_4_) and sodium dihydrogen phosphate (NaH_2_PO_4_) used for preparing simulated intestinal fluid (phosphate buffer) were obtained from Daejung Chemicals & Metals Co., Ltd., South Korea. FLX hydrochloride was purchased from Sigma Aldrich Inc., St. Louis, MO, USA. Hydrochloric acid was purchased from BDH Laboratory Supplies, Poole, Bh15 1TD, England. Potassium chromate (K_2_CrO_4_), potassium dichromate (K_2_Cr_2_O_7_), and mercuric chloride (HgCl_2_) used for the Golgi-Cox solution were purchased from Merck KGaA, Darmstadt, Germany. The ELISA kit used for plasma corticosterone estimation was purchased from Biomatik, Ontario, Canada.

### 2.2. Plant Nomenclature

  Name: *Mallotus oppositifolius* (Geiseler) Müll. Arg.
  Family: Euphorbiaceae.
  Local names: “Sroti” in Ewe, “Anyanforowa” in Asante, and “Sratadua” in Fanti.

The plant name was checked at https://wfoplantlist.org/plant-list.

### 2.3. Plant Collection and Extraction

Leaves of *M. oppositifolius* were harvested in August 2017, from the Center for Plant Medicine Research's (CPMR) Arboretum, Mampong-Akuapem, Ghana (5° 55′ 06.6″ N, 0° 07′57′57 W), authenticated by a botanist from the Plant Development Department at CPMR and assigned a Voucher Specimen Number CPMR 314/17. The cold maceration and rotary evaporation method used was as described by Koomson et al., [28]. Briefly, 3 kg of powdered leaves of *M. oppositifolius* was cold-macerated with ethanol (70% v/v) followed by rotary evaporation into a concentrate at 60°C under reduced pressure. The concentrate was then lyophilized (FreeZone 4.5, Labconco, Kansas City, MO, USA) to form a sticky gummy solid mass (1.05% yield) of extract (MOE) and stored in a desiccator until use.

Our laboratory conducted HPLC, gas chromatography, and mass spectrometric analyses on the plant extract, leading to the identification of various chemical constituents in the extract [29]. Thus, these analytical procedures were not repeated in this present study.

### 2.4. Determination of Maximum Wavelength of Absorption in 0.1 N HCl (pH 1.2) and Phosphate Buffer pH 4.5 and 6.8

To estimate the drug content and release rate of the *M.* formulations in gastric and intestinal medium, the maximum wavelength of absorption of the crude extract was determined spectrophotometrically. A 100 μg/mL solution of *M. oppositifolius* was prepared by dissolving 10 mg of the freeze-dried extract in 0.1 N HCl in a 100-mL volumetric flask. Dilutions of the solution were then made to obtain several concentrations (1 μg/mL −20 μg/mL). The various diluted solutions were scanned using a UV–vis spectrophotometer to obtain the maximum wavelength of absorption. The same procedure was repeated for the freeze-dried extract (crude extract) in phosphate buffer pH 4.5 and 6.8 to obtain the maximum wavelength of absorption [24].

### 2.5. Determination of the Calibration Curve of Extract at pH 1.2, 4.5, and 6.8

Exactly 100 mg of the extract was taken and dissolved in 100 mL of 0.1 N hydrochloric acid (HCl) to obtain 1000 μg/mL stock solution. From this stock solution, dilution with 0.1 N HCI was made to obtain concentrations of 5, 10, 15, 20, 25, 30, 35, and 40 μg/mL, respectively. The absorbance was measured at 295 nm using 0.1 N hydrochloric acid as blank. The same procedure was repeated for phosphate buffer pH 4.5 and 6.8, and the absorbance was taken at 294 nm and 292 nm, respectively.

### 2.6. Drug (Extract) Content Test

Granules from ten capsules of each formulation of *M. oppositifolius* was dissolved in 100 mL 0.1 N HCl separately. Each solution was filtered through Whatman filter paper No. 1 and diluted 100 times after which it was analyzed spectrophotometrically at the maximum wavelength previously determined [30, 31].

### 2.7. Weight Uniformity Test

The weight of each of 10 filled capsules of *M. oppositifolius* extract formulation was determined using an analytical balance. The capsule was then carefully opened, and all the content was removed totally. The empty shells were weighed. The difference between the weight of the initial filled capsules and empty shell was calculated. The mean weight of the 10 capsules was calculated, and the percentage deviations from the mean were determined [32].

### 2.8. Determination of Flow Properties of Formulated Extract

The flow properties of MOE granules were determined to predict the uniformity of weight of formulations and accuracy of dosing. In this study, we evaluated flow properties using bulk density, tapped density, Hausner's ratio, Carr's index, and the angle of repose as previously described [33]. Approximately 10 g of each granule formulation was poured into a 100-mL measuring cylinder. The initial volume known as the fluff volume (*V*_*o*_) was noted. The 100-mL measuring cylinder was then tapped 50 times, and the final volume known as the tapped volume (*V*_*f*_) was also noted. Carr's index and Hausner's ratio were then calculated.

The fixed height method was used to determine the angle of repose. The angle of repose was determined by weighing 3 g of the MOE granules into a funnel clamped to a retort stand with its tip 10 cm from the horizontal surface. The MOE granules were allowed to flow freely onto the horizontal surface, until the height and base of the cone was completed. The radius of the cone was used to calculate the angle of repose [34].

(1) $$\begin{array}{c} \text{Tapped density}=\frac{\text{Weight of MOE granules}}{\text{Tapped volume}}, \end{array}$$

(2) $$\begin{array}{c} \text{Bulk density}=\frac{\text{Weight of MOE }g\text{ranules}}{\text{Bulk volume}}, \end{array}$$

(3) $$\begin{array}{c} \text{Hausner ratio}=\frac{\text{Tapped density}}{\text{Bulk density}}, \end{array}$$

(4) $$\begin{array}{c} {\text{Carr}}^{\prime }s\text{ index}=\frac{\text{Tapped density}-\text{Bulk density}}{\text{Tapped density}}\times 100, \end{array}$$

(5) $$\begin{array}{c} \text{Angle of repose }\left(\theta \right)={\text{Tan}}^{-1}\left(\frac{\text{Height}}{\text{Radius}}\right). \end{array}$$

### 2.9. Preparation of Capsules

Immediate-release granules of *M. oppositifolius* (MOE-IR) was prepared using the wet granulation method as previously described by Shanmugam [35]. Previous reports by Kukuia [15] showed that the crude extract demonstrated an antidepressant activity at doses 10, 30, and 100 mg/kg, and this was the basis for the dose selected for formulation in this study. Concentration of sodium carboxymethyl cellulose 5% w/w and microcrystalline cellulose 10% w/w was selected from their working ranges [36]. Freeze-dried extract of *M. oppositifolius*, sodium carboxymethyl cellulose (NaCMc), lactose monohydrate, and the selected excipients were weighed accurately according to proportions in Table 1 and blended for 15 min by geometric dilution. The wet granulation method was employed as indicated above. Briefly, a portion of MCC was weighed as indicated in Table 1 and dispersed *d* in 10 mL of distilled water as granulating fluid and mixed with the powder blend gradually until a compact damp mass was formed. The damp mass was passed through Sieve No. 22 and oven-dried at 40°C for 40 min, and the capsules containing ∼350-mg granules each were subsequently prepared [32].

### 2.10. Disintegration Test

Disintegration or the breaking apart of oral pharmaceutical formulations is essential for the subsequent dissolution to take place. Gastrointestinal drug absorption of solid dosage forms can only occur after disintegration has taken place. To evaluate whether the formulation will be able to break apart and thus provide a wider surface area for dissolution, the disintegration test was carried out. With the use of an Erweka disintegration device, we followed the protocol given by the British Pharmacopoeia (QC-21, Disintegration Test System, Hanson Research, USA). Distilled water was used as the disintegrating medium. The bath in the disintegration apparatus was filled with water, and the temperature was maintained at 37°C ± 0.5°C. A capsule was placed into each of the six tubes of the basket rack. A disc was placed on each capsule to prevent it from floating. Disintegration time was determined and recorded. The procedure was done in triplicate for each formulation [24, 37].

### 2.11. Dissolution Test for Formulated Capsules

Dissolution of pharmaceutical products in the gastrointestinal tract is critical for predicting the bioavailability necessary for optimum therapeutic efficacy. The release of the *Mallotus oppositifolius* formulations was monitored using a USP Dissolution Tester Apparatus II (Hansen Research Corp., USA) with the paddle speed set at 100 rpm and a temperature of 37°C ± 0.5°C. Dissolution was carried out separately in different media of pH 1.2, 4.5, and 6.8 for an hour for each of the formulations and crude extract. About 10 mL aliquot was taken at 15-min intervals [15, 30, 45, 60] for 1 h and was replaced with fresh dissolution medium. The collected samples were filtered, diluted with 0.1 N HCl, and analyzed at 295 nm. The procedure was repeated at pH 4.5 (294 nm) and 6.8 (292 nm) separately [37, 38].

### 2.12. *In Vitro* Antioxidant Assay

The contributory role of oxidative stress under neuropsychiatric conditions such as depression and the beneficial effects of antioxidants is clearly delineated. Herein, we evaluated the *in vitro* antioxidant activity of the MOE crude and MOE-IR using the 1,1-diphenyl-2-picryl-hydrazyl (DPPH) assay as described by Nwaehujor et al. (2014). About 2 mL each of MOE crude and MOE-IR at 10–100 μg/mL was mixed with 1 mL of 0.5 mM DPPH dissolved in methanol, followed by incubation for 30 min at room temperature in the dark. Optical densities were measured at 517 nm using a spectrophotometer. Each sample was run three times, and hydrogen scavenging activity was calculated as indicated below:

(6) $$\begin{array}{c} \%\text{ Antioxidant Activity}=\left(\frac{\text{Abs}\left(\text{control}\right)-\text{Abs}\left(\text{sample}\right)}{\text{Abs}\left(\text{control}\right)}\right)\times 100, \end{array}$$

where sample = 2 mL extract + 1 mL methanol, and control = 2 mL methanol + 1 mL 0.5 mM DPPH solution.

### 2.13. Experimental Animals and Housing

Male Institute of Cancer Research (ICR) mice weighing 20–25 g (aged 6 weeks) were purchased from CPMR, Mampong, Eastern Region of Ghana. Mice were housed in steel cages (34 × 47 × 18 cm^3^) with wood shavings as beddings and kept at the Animal House of the Department of Medical Microbiology, University of Ghana Medical School, University of Ghana. They were fed with normal standard rodent chow (GAFCO, Ghana) and given water *ad libitum*. Ethical clearance for the study was obtained from the Ethical and Protocol Review Committee of the College of Health Sciences, University of Ghana, and assigned Identification Number: CHS-Et/M.3-P 4.3/2021-2022. All procedures employed in the work followed the ARRIVE guidelines and were in conformity with the National Institute of Health Guidelines for the Care and Use of Laboratory Animals [39].

### 2.14. Experimental Design

**Figure 1:**
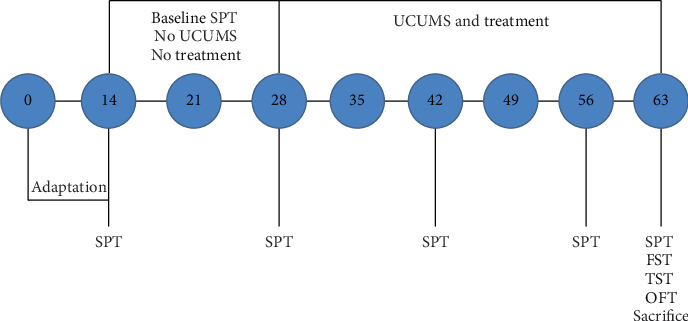
Experimental design.

Mice were randomly grouped (*n* = 8) through minimization. Briefly, each group was assigned equal number of mice based on their body weight. We ensured that animals of the same weight were not kept in the same group to achieve some uniformity in weight distribution across the groups. Sample size was chosen based on previous studies where similar numbers gave reliable results [14]. Grouped mice were taken through unpredictable chronic mild stress (UCMS) along with sucrose preference test (SPT) and weight evaluation while receiving daily treatment. About 24 h after treatment discontinuation test, forced swim test (FST), tail suspension test (TST), and open field test (OFT) were conducted. Assessment of all behavioral effects of treatment was done by a trained blinded observer who was unaware of treatments given to each group of mice. After these behavioral experiments, blood and brains were taken to evaluate corticosterone levels and hippocampal neuronal dendritic spine densities, respectively. Refer to Figure 1.

### 2.15. UCMS

The method involves subjecting an animal at unpredictable times a series of minor-intensity stressors over several weeks, which culminates to the development of several behavioral alterations in a large majority of animals, including the loss of pleasure (anhedonia) and apathy. These behavioral changes, with attendant alterations in certain neural and endocrine variables, mimic the symptoms of major depressive disorder and, just as with the clinical condition, can be reversed by a wide range of antidepressant drugs with different mechanisms of action [40].

The method used was as previously described by Adongo [5].^.^The randomly grouped mice were assigned to six different groups (*n* = 8) and allowed to acclimatize to the experimental environment for 2 weeks before the commencement of the test. Prior to the UCMS test, baseline sucrose consumption by each mouse was measured for 2 weeks. At the beginning of the third week, all groups, except Group 1 (vehicle nonstressed, VEH-NS), were subjected to UCMS procedure for 5 weeks. UCMS procedure involved subjecting mice to a daily randomized series of mild environmental and social stressors such as food deprivation for 24 h, water deprivation for 24 h, tail pinch for 1 min, physical restraint for 2 h (plastic bottles), cold swimming for 5 min, soiled cage for 24 h, and overnight illumination or cage tilting at 45° for 7 h. The stressors were continuously varied to make them unpredictable. These ensured mice were unable to anticipate, learn, and pre-empt the next stressor that will be applied. During the 5 weeks, Group 2 received normal saline, representing the vehicle-stressed group (VEH-S, 10 mL/kg); Group 3: FLX (20 mg/kg, p.o) dissolved in saline; and Groups 4–6: MOE-IR capsules (10, 30, and 100 mg/kg, *p.o*) suspended in saline. The doses of MOE used were based on a previous study by Kukuia et al. [14]. Treatment-induced changes in sucrose preference and weight were assessed every week for the 5 weeks. Stress-induced depression-like state was estimated through reduction in sucrose preference, which was interpreted as the loss of interest (anhedonia) and weight changes of mice.

### 2.16. SPT

As a reward-based test, the SPT is used in evaluating anhedonia. Anhedonia, or the decreased ability to experience pleasurable things, is a core symptom of depression. Rodents have an innate appetite for sweet foods, and hence, reduced preference for sweet solution in the SPT represents anhedonia, while this reduction can be reversed by treatment with antidepressants [41]. The procedure was done as described by Shen et al. [42]. Mice in each group were individually housed in a cage and made to adapt to drinking from two bottles, each containing 1% sucrose solution for 24 h. After this, one bottle was replaced with water, while the other still contained 1% sucrose solution for the next 24 h. Positions of the two bottles were switched every 12 h to avoid the influence of bottle position preference effects, and there was freedom to access any of the two bottles. This is known as the habituation phase. After the habituation phase, the animals were deprived of food and water for 23 h to prevent the influence of diet on sucrose preference. Two preweighed bottles were then simultaneously put into the cage, one containing 100 mL of 1% sucrose and the other containing 100 mL of pure water, and the animals were allowed to drink from these bottles for 1 h. Sucrose preference was calculated as indicated below:

(7) $$\begin{array}{c} \text{Sucrose preference}=\frac{\text{Sucrose intake }\left(g\right)}{\left(\text{Sucrose intake}+\text{Water intake}\right)\;\left(g\right)}\times 100\%, \end{array}$$

### 2.17. FST and TST

Following 24 h of treatment cessation in the UCMS-SPT, we assessed whether the antidepressant-like effect of MOE-IR will be sustained using the FST [43] and TST [44].

In the FST, mice were allowed to swim in an open inescapable cylindrical container (diameter 10 cm, height 25 cm), containing water at a depth of 19 cm and a temperature of 25 ± 1°C. The duration of immobility and swimming and climbing time were recorded with a video recorder (Sony 4K Handy Cam, FDR-A × 100E) in a 6-min session and analyzed with a video-tracking system by an observer blinded to the treatment procedure. Mouse was deemed immobile when it ceased struggling to escape and remained floating on the water, showing movements only necessary to keep its head above water. Mice were considered to be swimming if they were actively moving their bodies in the water beyond what was required to keep their heads above water, and climbing if their forepaws were actively moving in and out of the water, typically directed toward the walls of the container. A reduction in immobility behavior was considered the indication of antidepressant-like effect.

Behavioral despair was measured in the TST. Each group of mice was suspended by the tail from a metal rod mounted 50 cm above the surface by fastening the tail to the rod with an adhesive tape. The test lasted for 6 min, and immobility, swinging, and curling behaviors were recorded using a video recorder (Sony 4K Handy Cam, FDR-A × 100E) and analyzed by a blinded observer. A mouse was considered to be immobile when it hung by its tail without engaging in any active behavior except for respiratory movements; swinging, when its paws were continuously moved in the vertical position while keeping its body straight and/or it moved its body from side to side; and curling when it engaged in active twisting movements of the entire body so that the tail part of the body is raised toward the head.

### 2.18. OFT

The OFT was used to assess the effect of treatment on locomotor activity based on a method described [45]. The open field box, which was placed on the floor of the experimental room, had dimensions of 60 × 60 × 25 cm^3^ with the central compartment measuring 20 × 20 cm^2^. Each was dimly lit. Each mouse was gently placed in the center and allowed to explore freely. The floor was wiped with 70% ethanol after every session to eliminate odors that might interfere with exploratory behavior of the next animal. The animal behavior was recorded using a video camera (Sony 4K Handy Cam, FDR-A × 100E) mounted to point at the top view of the box. The number of lines crossed by each mouse was assessed manually by an observer blinded to the treatments given. An animal was deemed to have crossed a line into another square when all paws cross the line. Locomotor activity was scored as total distance traveled (marked by the number of lines crossed) for a 6-min period.

### 2.19. Blood and Brain Sample Collection

Immediately after the last behavioral assessment, mice were anesthetized according to the method described by Kukuia et al. [46]. Mice were sacrificed after the last day of behavioral test by anesthetizing them using diethyl ether in an anesthesia induction chamber, and blood was collected through direct cardiac puncture into EDTA-coated tubes and the plasma separated by high-speed centrifugation (4000 g × 20 min) [45]. Samples were taken into Eppendorf tubes and stored at (−20°C) until use. The whole brains were rapidly separated from the skull and four [4] from each group stored at −80°C for histological examination.

### 2.20. Plasma Corticosterone Estimation

Serum corticosterone was estimated using a mouse CORT (BIOMATIK, Ontario, Canada) ELISA kit following the manufacturer's instructions. Approximately 50 μL of standard and samples was added in each well followed by 50 μL of detection antibody solution. The plate was incubated for 45 min at 37°C. Each well was drained and washed with the wash solution. This was followed by addition of 100 μL horseradish peroxidase (HRP) conjugate immediately to each well and incubated at 37°C for 30 min after which each well was washed five [5] times with wash buffer. After washing, 90 μL of 3,3′,5,5′-tetramethylbenzidine (TMB) substrate was added to each well and incubated for 20 min at 37°C whiles protecting from light. The reaction was stopped by 50 μL stop solution added to each well and mixed thoroughly. The optical density of colored product in each well was measured by means of a microplate reader (MULTISKAN EX, Thermo Fisher Scientific).

### 2.21. Brain Histology

#### 2.21.1. Preparation of Golgi-Cox Solution

This was done according to the method used by Kukuia [29]. The impregnation solution (Golgi Cox) was prepared from a mixture of potassium dichromate (K_2_Cr_2_O_4_), mercuric chloride (HgCl_2_), and potassium chromate (KCrO_4_) each at 5% w/v at a ratio of 5:5:4, respectively. The resulting solution was stored in dark for 5 days until use.

#### 2.21.2. Tissue Collection and Preservation

Brain tissues of the mice were severed quickly from the cranium after transcardially perfusing with cold normal saline. The tissues were impregnated with Golgi-Cox solution contained in 40-mL bottles for a full day. Following this, the solutions were discarded, and the containers were filled with fresh Golgi-Cox solution and the samples returned to the container. The brain samples were then stored in the dark for fourteen 14 days. After the fourteenth day, the brains removed from the solution were blotted and transferred into the 30% w/v sucrose. These brain samples were stored in the refrigerator (−80°C) until sectioning were done at 50 μm using a microtome. The area of interest was the dentate gyrus (DG) of the hippocampus.

#### 2.21.3. Tissue Processing and Sectioning

Each brain sample was divided into three coronal sections, placed in histological cassettes (Rotilabor embedding cassettes; K114.1, Carl Roth GmbH, Germany), and passed through ethanol at varying concentrations of 70% for an hour, 95% for one and a half hour, 100% for two hours, and finally another 100% for two hours. They were then put in molten paraffin wax for 3 hours and refrigerated until sectioning. The blocks were sectioned at 50 μm using a Leica RM 2235 manual microtome and placed on water.

#### 2.21.4. Gelatinization of Slides

Slides for the sections were soaked in gelatin solution while avoiding the formation of bubbles. This was followed by oven drying at 37°C overnight. The sections were picked from the surface of water and mounted on the slides coated in gelatin. They were transferred to slide racks and allowed to dry for 3 days in the dark.

#### 2.21.5. Color Development

Dewaxing of the slides was done by dipping them in xylene for 2 min followed by 2-min dipping in 100% and 5-min dipping in 50% ethanol. The slides were transferred to 3:1 ammonia in darkness for 8 min followed by a wash with double distilled water for 5 min. The slides were then immersed in 1% sodium thiosulfate solution for 5 min to ensure that the stain is fixed before a 1-min wash in distilled water. This was followed by incubation in Mallory stain C 5% as a counterstain. Slides were dehydrated for 5 min each in an ethanol series of 70%, 95%, and 100% (twice). Finally, the slides were transferred to fresh xylene for 2 min in the dark and allowed to dry under a fume chamber for 3 days before microscopic examination.

#### 2.21.6. Neuronal Count and Dendritic Spine Density Analysis

Observation of slides was done under a microscope (Leica Galen III-1154XV) at × 100 and × 400 magnifications. Using a coupled device eye piece (JVC, Tokyo, Japan, and MBF CX9000) linked to the microscope using Image-Pro Plus 5.1.1 (Media Cybernetics, Silver Spring, USA) software, hippocampal images were obtained, focusing on the granular layer of the DG.

For the dendritic spine assessment, sections of labeled neurons were viewed under the light microscope with an oil immersion (100×) and scanned at 1-μm intervals. Assessment of dendritic length and spine features was done as previously described by Kukuia et al. [29]. We focused on apical dendrites of the granular cells that were clearly attached to the cell body of interest within the frame of 75–200 μm range. Briefly, three separate coronal sections per mouse were analyzed. Z-stack images of the Golgi spines were processed with ImageJ software (Fiji Version 1.53c, NIH) and imported into the publicly available Reconstruct software (https://synapses.clm.utexas.ed) as series images. These images were calibrated and processed for the analysis of the length and width of the dendritic spines. A dendritic segment for all sections had to be 10 μm in length uninterrupted. The data were imported and computed in a spreadsheet. Spine density was calculated by quantifying the number of spines per dendritic segment and normalized to 10 μm of dendrite length. The morphological characteristics of the spines were classified as mushroom, filopodia, stubby, long thin, thin, and branched, based on the length, width, and length-to-width ratio of the spines as previously described [29]. The results were then exported to GraphPad Prism for statistical analysis.

### 2.22. Statistical Analysis

All statistical analyses and graphical representations were done using GraphPad Prism 8.0.2 (GraphPad software, San Diego, CA, USA). Values were presented as the mean ± standard error of the mean. Variations in means were determined using either one-way or repeated-measures two-way analysis of variance (ANOVA) followed by Tukey's *post hoc* multiple comparisons test or the Bonferroni test. In all tests, *p* < 0.05 was considered statistically significant.

## 3. Results

### 3.1. Content Analysis of *M. oppositifolius* Formulation

We present the results of drug content analysis of two formulations of the IR formulations of MOE (MOE-IR F_1_ and F_2_). The content for both formulations was between 91.46% and 105.27%. However, we observed that MOE-IR F_1_ had a higher content (103.59%–105.27%) when compared to MOE-IR F_2_ (91.46%–102.91%). Notwithstanding, all values obtained after content analysis were within the acceptable U.S. pharmacopoeial range. Refer to Table 2.

### 3.2. Powder Flowability Properties of *M. oppositifolius* Extract

The present study evaluated the flowability properties of the formulated extract of *M. oppositifolius*. Parameters measured include the angle of repose, Hausner's ratio, and Carr's index. We show that for both formulations (MOE-IR F_1_ and F_2_), as dose increases, there is a corresponding increase in the angle of repose, Hausner's ratio, and Carr's index (Table 3). Although all parameters measured fell within the acceptable criteria for powders with good flow properties, MOE-IR F_2_ had slightly higher values when compared to MOE-IR F_1_ (Table 3).

### 3.3. Capsule Uniformity of Weight

The formulations had good physical properties with average capsule weight range being 350–354.5 mg and % deviation < 7.5%. These were within the acceptable compendial limits [47].

### 3.4. Disintegration Time

Before the active constituents of solid dosage forms are absorbed, disintegration, which can influence the rate of absorption, usually happens. In this study, we present the disintegration times for the two IR formulations of MOE. We found that the mean disintegration time for MOE-IR F_1_ (10–100 mg) was 8.47 min, while that of MOE-IR F_2_ (10–100 mg) was 8.44 min Table 4.

### 3.5. Drug Release Profiles

Release profiles of the IR formulations MOE at different pH were compared with that of crude extract (MOE crude) and presented as time-course graphs. A two-way ANOVA was conducted to compare the differences in dissolution profiles of the two formulations and the crude extract at different pH that mimics various parts of the gastrointestinal tracts where drug release and absorption take place.

### 3.6. pH 1.2

**Figure 2:**
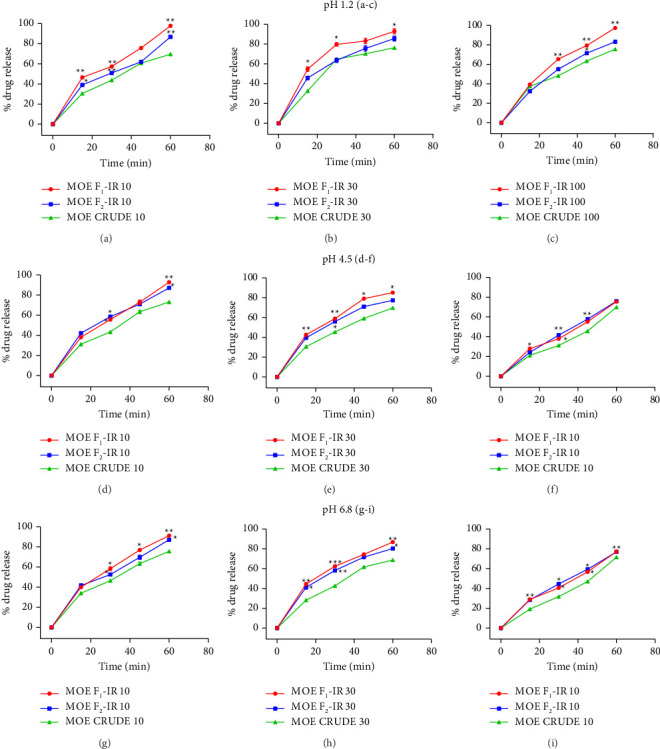
Dissolution profiles of extracts in acidic pH 1.2, 4.6, and 6.8, presented as time-course graphs. (a) MOE-IR F_1_ (10 mg), MOE-IR F_2_ (10 mg), and MOE crude (10 mg); (b) MOE-IR F_1_ (30 mg), MOE-IR F_2_ (30 mg), and MOE crude (30 mg); (c) MOE-IR F_1_ (100 mg), MOE-IR F_2_ (100 mg), and MOE crude (100 mg) at pH 1.2. (d) MOE-IR F_1_ (10 mg), MOE-IR F_2_ (10 mg), and MOE crude (10 mg); (e) MOE-IR F_1_ (30 mg), MOE-IR F_2_ (30 mg), and MOE crude (30 mg); (f) MOE-IR F_1_ (100 mg), MOE-IR F_2_ (100 mg), and MOE crude (100 mg) at pH 4.6. (g) MOE-IR F_1_ (10 mg), MOE-IR F_2_ (10 mg), and MOE crude (10 mg); (h) MOE-IR F_1_ (30 mg), MOE-IR F_2_ (30 mg), and MOE crude (30 mg); (i) MOE-IR F_1_ (100 mg), MOE-IR F_2_ (100 mg), and MOE crude (100 mg) at pH 6.8. Data are presented as mean ± S.E.M (*n* = 3). ^∗∗^*p* < 0.01 and ^∗^*p* < 0.05 (repeated measure two-way ANOVA followed by the Bonferroni post hoc test).

MOE-IR-F1 at all doses exhibited a significantly higher drug release profile across the time points compared with the crude extract. At a pH of 1.2, MOE-IR F_1_ (10, 30, 100 mg) exhibited a drug release of 97.85 ± 1.81%, 92.93 ± 4.51%, and 74.23 ± 1.62%, respectively (Figures 2(a), 2(b), 2(c)). In contrast, MOE-IR F_2_ (10, 30, 100 mg) gave a release profile of 86.8 ± 1.26% %, 85.56 ± 4.38%, and 83.2% ± 2.82, while the MOE crude (10, 30, 100 mg) exhibited a drug release of 75.2 ± 1.36%, 79.71 ± 0.74%, 74.33 ± 1.62%, respectively (Figures 2(a), 2(b), 2(c)). In comparing release profiles at different doses, we found that at 10 mg, MOE-IR F_1_ was significantly (*F*_4,8_ = 3852; *p* < 0.0001) greater than MOE-IR F_2_ and the crude at the same dose over the 1-h period (Figure 2(a)). Similarly, at 30 mg, MOE-IR F_1_ (*F*_4,8_ = 1546; *p* < 0.0001) performed better than MOE-IR F_2_ and the crude extract at similar doses (Figure 2(b)). Just as previously, at 100 mg, MOE-IR F_1_ (*F*_4,8_ = 24,060; *p* < 0.0001) had a greater release profile than MOE-IR F_2_ and the crude extract (Figure 2(c)). In summary, it appears that in an acidic medium, the MOE-IR F_1_ released a greater amount of the extract within 1 hour, followed by MOE-IR F_2_ and then MOE crude.

### 3.7. pH 4.5

At pH 4.5, a similar pattern of results obtained at pH 1.2 was observed. MOE-IR F1 consistently showed the highest release profile when compared to both MOE-IR F2 and crude extract at all doses tested (Figures 2(d), 2(e), 2(f)). In this weakly acidic medium of pH 4.5, MOE-IR F1 (10, 30, and 100 mg) released 92.8 ± 1.5%, 85.1 ± 1.0%, and 75.5 ± 2.14% within one hour, respectively (Figures 2(d), 2(e), 2(f)). On the other hand, MOE-IR F2 (10, 30, and 100 mg) exhibited a release profile of 87.2 ± 1.4%, 77.4 ± 2.4%, and 76 ± 1.4%, respectively, while MOE crude (10, 30, and 100 mg) released 73.2 ± 2.5%, 69.7 ± 1.7%, and 69.4 ± 1.4% within one hour, respectively (Figures 2(d), 2(e), 2(f)). At 10 mg (*F*_4,8_ = 4689; *p* < 0.0001), 30 mg (*F*_4,8_ = 2221; *p* < 0.0001), and 100 mg (*F*_4,8_ = 3069; *p* < 0.0001), MOE-IR F1 exhibited significantly higher release profiles than MOE-IR F2 and the crude extract. Taken together, MOE-IR F1 released greater amount of extract, followed by MOE-IR F2 and then MOE crude as shown in Figures 2(d), 2(e), 2(f).

### 3.8. pH 6.8

At pH 6.8, dissolution across different time points was found to be significantly different between formulations tested. From our assessment of the release profiles of the extract at this pH, MOE-IR F_1_ (10, 30, 100 mg) gave 91.2 ± 2.2%, 86.8 ± 1.0%, and 77.1 ± 1.57%, respectively; MOE-IR F_2_ (10, 30, and 100 mg) gave 87.1 ± 1.5%, 80.4 ± 2.4%, and 77 ± 1.3%, respectively; MOE crude (10, 30, and 100 mg) gave 73.2 ± 2.5%, 69.7 ± 1.7%, and 69.9 ± 1.4%, respectively, within an hour (Figures 2(g), 2(h), 2(i)). Analysis of comparative doses showed that at 10 mg, MOE-IR F_1_ had a significantly (*F*_4,8_ = 1443; *p* < 0.0001) higher release profile than MOE-IR F_2_ and crude (Figure 2(g)). Similar trends were observed where MOE-IR F_1_ had a higher release profile than the other formulations and MOE crude at 30 mg (*F*_4,8_ = 1466; *p* < 0.0001; Figure 2(h)) and 100 mg (*F*_4,8_ = 2580; *p* < 0.0001, Figure 2(i)). Taken together, MOE-IR F_1_ demonstrated a greater release profile than MOE-IR F_2_ and MOE crude (Figures 2(g), 2(h), 2(i)).

### 3.9. DPPH Scavenging Activity

**Figure 3:**
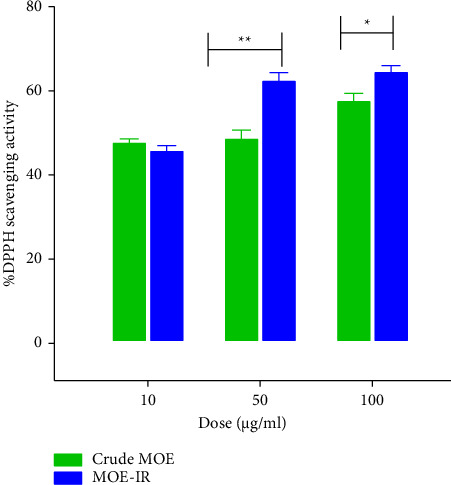
Antioxidant activity of immediate-release *Mallotus oppositifolius* formulation (MOE-IR 10, 50, 100 μg/mL) and crude *Mallotus oppositifolius* extract (MOE crude 10, 50, 100 μg/mL). Data are presented as mean ± SEM (*n* = 3). ^∗^*p* < 0.05 and ^∗∗^*p* < 0.01 (two-way ANOVA followed by the Bonferroni post hoc test).

Antioxidant activity of MOE-IR (the best formulation selected from the two) and MOE crude (Figure 3) showed that MOE-IR exhibited a higher DPPH scavenging activity when compared to MOE crude. The percentage antioxidant activity of MOE-IR increased from ∼48% at a concentration of 10 μg/mL to ∼65% at 100 μg/mL. In contrast, the same dose of MOE crude exhibited an increase of ∼47% to ∼57% DPPH scavenging activity.

### 3.10. Effects of Formulations on Body Weight

**Figure 4:**
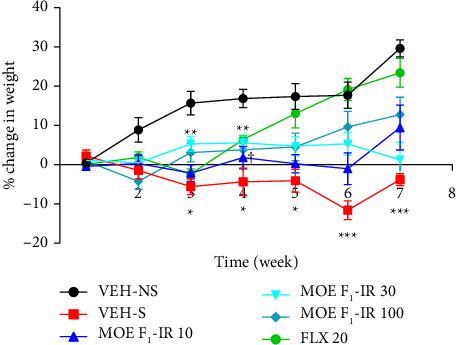
Effects of MOE F_1_-IR (10, 30, and 100 mg/kg) and fluoxetine 20 mg/kg on weight changes. Data are presented as mean ± SEM (*n* = 6–8) on a time-course curve. Significantly different from the vehicle-treated nonstressed group: ^∗∗∗^*p* < 0.001, ^∗∗^*p* < 0.01, and ^∗^*p* < 0.05 (repeated measure two-way ANOVA followed by the Bonferroni post hoc test).

Depression and certain antidepressants can adversely affect body weight. Thus, the effect of MOE-IR treatment on weight of mice was assessed. From the time-course graph, weights of the mice were similar prior to the UCMS exposure. After 2 weeks of exposure to stressors, a loss in body weight was observed across all stressed groups. From the third [3] week when treatment began, both MOE-IR and FLX groups exhibited a gradual reversal in weight loss, while VEH-S mice showed further weight loss throughout the study. Unsurprisingly, the VEH-NS mice showed a consistent increase in weight Figure 4.

### 3.11. UCMS-SPT

**Figure 5:**
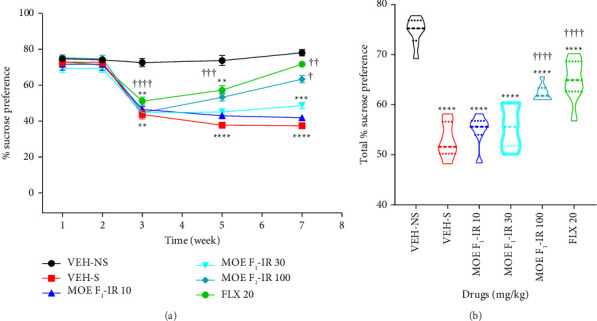
Effects of MOE F_1_-IR (10, 30, and 100 mg/kg) and fluoxetine 20 mg/kg treatment on the sucrose preference of mice subjected to chronic unpredictable mild stress. Data are represented as both (a) a time-course curve and (b) the mean ± SEM of their AUCs (*n* = 6–8). Significantly different from the vehicle-treated no stress group: ^∗∗∗∗^*p* < 0.0001, ^∗∗∗^*p* < 0.001, and ^∗∗^*p* < 0.01; (repeated-measures two-way ANOVA followed by the Bonferroni post hoc test); significantly different from the stress group ^††††^*p* < 0.0001 and ^†††^*p* < 0.000 (repeated-measures two-way ANOVA followed by the Bonferroni post hoc test). Significantly different from the vehicle-treated no stress group: ^∗∗∗∗^*p* < 0.0001 (one-way ANOVA followed by Tukey's multiple comparison test). ^††††^*p* < 0.0001, significantly different from stress group (one-way ANOVA followed by Tukey's test).

Due to its similarities with clinical depression, the UCMS together with SPT was used to assess the antidepressant effect of the IR formulation of MOE. The results are presented as the time-course graphs of sucrose preference (Figure 5(a)) together with their areas under the curve, presented as violin plots (Figure 5(b)). We observed that in the first 2 weeks, sucrose consumption was relatively the same across all groups. However, after introducing the depression-inducing stressors from the third week, sucrose consumption significantly (*F*_5,39_ = 45.74; *p* < 0.0001) declined in all stressed groups. In contrast, the VEH-NS mice did not exhibit any decrease in sucrose preference. Unsurprisingly, the stress vehicle (VEH-S) groups exhibited stress-induced anhedonia until the end of the 7-week experiment. In comparison with the VEH-S, the stressed-induced decline in sucrose preference was reversed by MOE-IR and FLX treatment from the fifth to seventh weeks (Figure 5(a)). Although sucrose preference in the stressed mice that received MOE-IR and FLX was significantly lower than that of the VEH-NS mice, this returned to parity by the last week of treatment (Figure 5(a)). In addition, we observed that the total percentage sucrose preference was significantly (*F*_5,39_ = 45; *p* < 0.0001) higher in mice treated with MOE-IR and FLX when compared to the VEH-S mice, but not as high as the VEH-NS group (Figure 5(b)).

### 3.12. FST and TST

**Figure 6:**
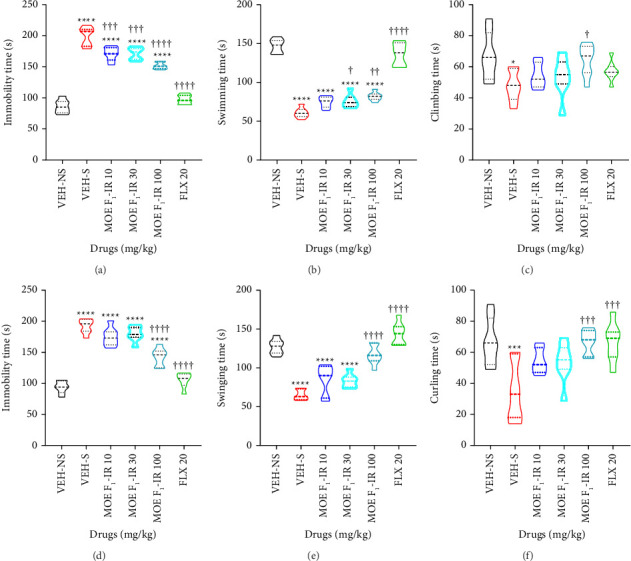
Effects of MOE F_1_-IR (10, 30, and 100 mg/kg), and fluoxetine 20 mg/kg treatment on (a) duration of immobility, (b) duration of swimming time, and (c) climbing score in the force swimming test (FST); effects of MOE F_1_-IR (10, 30, and 100 mg/kg), and fluoxetine 20 mg/kg treatment on (d) duration of immobility, (e) duration of swinging, and (f) duration of curling in the tail suspension test (FST). Data are presented as group means ± SEM (*n* = 6–8). Significantly different from the vehicle-treated no stress group: ^∗∗∗∗^*p* < 0.0001 and ^∗^*p* < 0.05; (one-way ANOVA followed by Tukey's post hoc test). ^††††^*p* < 0.0001, ^†††^*p* < 0.0001, ^††^*p* < 0.001, and ^†^*p* < 0.05; significantly different from the stress group (one-way ANOVA followed by Tukey's multiple comparison test).

Approximately 24 h after treatment discontinuation in the UCMS test, we used the FST and TST to evaluate the sustained antidepressant-like effect of MOE-IR (Figures 6(a), 6(b), 6(c)). In the FST, treatment groups exhibited a significantly (*F*_5,36_ = 132.5; *p* < 0.0001) reduced immobility time (Figure 6(a)). Post hoc analyses revealed that the stressed group (VES-S) had a remarkably increased immobility time (*p* < 0.0001) in comparison with the VEH-NS group, given credence to the UCMS model's success in inducing depressive-like states. FLX, just as MOE-IR (10, 30, 100 mg/kg) significantly (*p* < 0.0001) reduced immobility time when compared to the stressed group (VES-S). Moreover, MOE-IR, just as FLX, significantly (*F*_5,36_ = 122.2; *p* < 0.0001) increased swimming behavior (Figure 6(b)). It is worth noting that although MOE-IR 10 mg/kg did not produce a significant difference (*p* > 0.05) in swimming time, MOE-IR 30 (*p* = 0.0310) and 100 mg/kg (*p* < 0.0012) induced an increase in swimming time in a dose-dependent fashion, respectively. Conversely, only MOE-IR 100 significantly (*p* = 0.0424) increased climbing behavior, when compared to the VEH-S. MOE-IR 10 and 30 mg/kg as well as FLX failed *p* > 0.05 to produce any significant change in the climbing score (Figure 6(c)).

The TST was used to validate the sustained antidepressant-like effect of MOE-IR observed in the FST. There were significant differences in immobility behavior among the treatment groups (*F*_5,36_ = 75.63; *p* < 0.0001) (Figures 6(d), 6(e), 6(f)). Here, we show that the VEH-S mice exhibited a significantly (*p* < 0.0001) greater immobility score but lower swinging and curling scores when compared to the VEH-NS. The stress-induced increase in immobility behavior was significantly (*p* < 0.0001) attenuated by MOE-IR (100 mg/kg) when compared to VEH-S, but the reversal was not comparable to the immobility status of VEH-NS mice (Figure 6(d)). No significant reduction in immobility time was observed with the MOE-IR 10 and 100 mg/kg doses. FLX reversed the stress-induced increase in immobility score to the level of VEH-NS (Figure 6(d)). Moreover, MOE-IR and FLX reversed the stressed-induced diminution in swinging (*F*_5,36_ = 40.68 *p* < 0.0001) (Figure 6(e)) and curling (*F*_5,36_ = 6.5 *p* < 0.0001) (Figure 6(f)).

### 3.13. OFT

**Figure 7:**
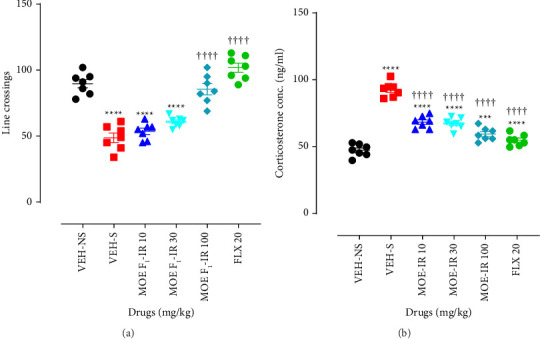
(a) Effects of MOE F_1_-IR (10, 30, and 100 mg/kg), and fluoxetine (20 mg/kg) treatment on line crossing in the open field test (OFT). (b) Effects of MOE F_1_-IR (10, 30, and 100 mg/kg) and fluoxetine (20 mg/kg) treatment on plasma corticosterone. Data are presented as group means ± SEM (*n* = 6–8). Significantly different from the vehicle-treated no stress group: ^∗∗∗∗^*p* < 0.0001 (one-way ANOVA followed by Tukey's post hoc test). ^††††^*p* < 0.0001, significantly different from stress (one-way ANOVA followed by Tukey's multiple comparison test).

Decreased immobility, which depicts the antidepressant effect, can be induced by drugs with psychostimulant effects, thus producing false-positive antidepressant-like results. Hence, to ascertain whether the antidepressant-like effects of MOE-IR were not due to any psychostimulant activity, we used the number of line crossings in the OFT to assess locomotor activity in mice. Our study revealed that VEH-S had fewer line crossings when compared to VEH-NS. Similarly, lower doses of MOE-IR (10 and 30 mg/kg) produced fewer line crossings (*p* < 0.0001) compared to VEH-NS, whereas the highest dose (MOE-IR 100 mg/kg) and FLX produced line crossings comparable to the VEH-NS (Figure 7(a)).

### 3.14. Plasma Corticosterone Estimation

Stress-induced depressive symptoms are usually characterized by increases in the plasma corticosterone concentration in mice. Herein, we assessed the effect of MOE-IR on the concentration of plasma corticosterone in stressed mice. The present study showed that there was a significant difference in plasma corticosterone levels among the groups (*F*_5,36_ = 76.30; *p* < 0.0001). VEH-S had significantly higher plasma corticosterone levels compared to the VEH-NS group (Figure 7(b)). The stress-associated rise in corticosterone levels was significantly (*p* < 0.0001) reduced by all MOE-IR doses (10, 30, and 100 mg/kg) and FLX FLX (20 mg/kg) when compared to VEH-S. Despite the reduction in the corticosterone levels, only FLX 20 mg/kg was able to bring it down to levels comparable to the nonstressed (VEH-NS) state *p* > 0.05 (Figure 7(b)).

### 3.15. Effect of Extract Formulation on Dentate Gyrus Dendritic Spine Densities

**Figure 8:**
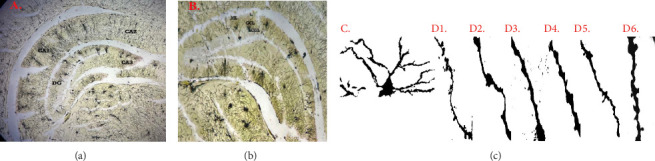
Representative photomicrographs of Golgi-Cox–stained brain showing (a) hippocampus with the CA1, CA2, CA3 and dentate gyrus (DG); (b) dentate gyrus revealing the molecular layer (ML), granular cell layer (GCL) with granular neurons and the sub-GCL (sGCL); (c) granular neuron of the dentate gyrus with its apical dendrites and spines (d1–d6). Representative dendritic spine images for vehicle nonstressed (VEH-NS); vehicle stressed (VEH-S); MOE-IR 10 mg/kg; MOE-IR 30 mg/kg; MOE-IR 100 mg/kg; and fluoxetine (FLX), respectively, × 100 magnification.

**Figure 9:**
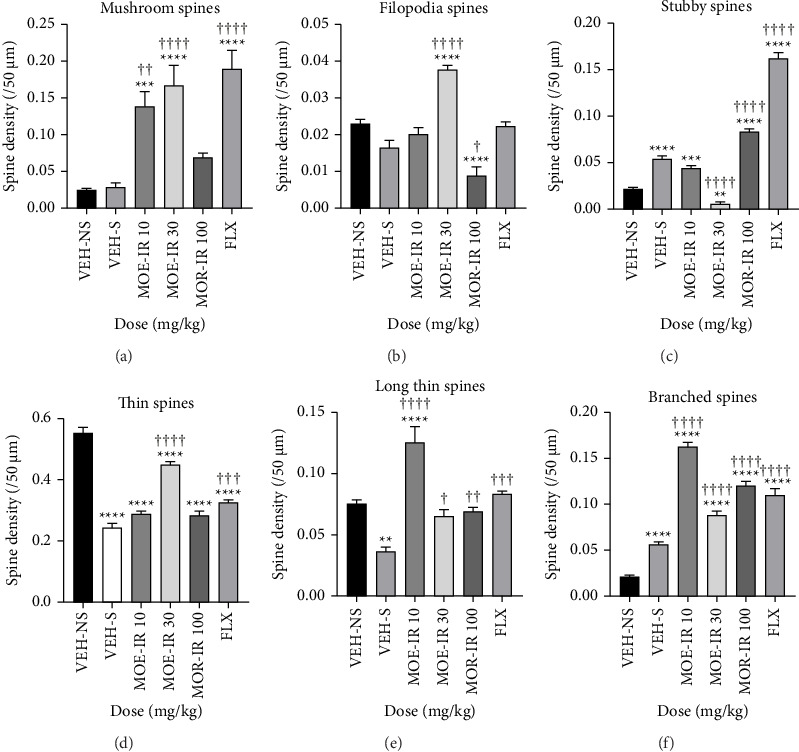
Effects of MOE F_1_-IR (10, 30, and 100 mg/kg) and fluoxetine (20 mg/kg) treatment on granular neuron apical dendritic spine density. Data are presented as group means of each spine type ± SEM.

We evaluated the effect of MOE-IR on the DG dendritic spine densities, focusing on the granular layer neurons and the apical dendrites attached to them (Table 5).  Figure 8(a) shows a representative photomicrograph of the CA1, CA2, and CA3 regions of hippocampus as well as the DG; Figure 8(b) shows the DG with the molecular layer (ML), granular cell layer (GCL) with granular neurons and the sub-GCM (sGCL); and Figure 8(c) presents a representative granular layer neuron with its apical dendrites and spines. Figures 8(d1), 8(d2), 8(d3), 8(d4), 8(d5), 8(d6) present the representative spine types on the apical dendrites. From our analysis, we observed that the percentage of thin dendritic spines was generally higher in all groups compared to the other spine types (Figure 9). It was also observed that the VEH-NS did not significantly alter spine densities. However, the MOE-IR group showed higher mushroom-, stubby-, thin-, long thin-, and branched-type spines when compared to the VEH-S group (Figure 9). Interestingly, only the lowest dose of MOE-IR increased the filopodia spines (Figure 9(b)).

## 4. Discussion

Although the safety and remarkable antidepressant efficacy of *M. oppositifolius* extract have been extensively investigated in animal models, an optimized formulation was prepared to enhance its efficacy, patients' convenience, and compliance [14, 15, 48, 49]. Thus, the current study explored the antidepressant-like effects of an IR formulation of MOE in mice. Herein, we show that MOE-IR exhibited antidepressant-like effects by reversing stress-induced anhedonia in the UCMS-SPT. The behavioral results were supported by a reduction in plasma corticosterone and increased DG dendritic spine density.

To ensure that the formulated extract had the required characteristics, we assessed flow properties, content and weight uniformity, disintegration, and dissolution profiles of various IR formulations of MOE and compared it to the crude extract of *M. oppositifolius*. The ease of flow of powders ensures reproducibility in capsule dosators, enhancing weight uniformity and the required consistency in the physical properties of the powder. The focus of powder flow investigations is to ensure that less cohesive powders with good flow properties are used for formulation. Free-flowing and less cohesive powders significantly influence consistency in weight uniformity and ultimately dosing accuracy. A Hausner ratio close to 1.2, an angle of repose close to 25°, is indicative of a less cohesive powder, suggesting a free-flowing powder, but Hausner's ratio > 1.6 and angles of repose above 40° suggest poor or unsatisfactory flow properties. Carr's compressibility index of < 15% has an excellent flow, that of 12%–16% has a good flow, that of 18%–21% a poor flow, and that of above 40% has a highly unsatisfactory flow. Generally, preformulation characteristics of *M. oppositifolius* formulations showed that the angle of repose, Hausner ratio, and Carr compressibility were all within the criteria indicative of a powder with good flow properties [33, 34]. However, the crude extract (MOE crude had fairly good flow properties). Aside the flow properties, quality control tests for drug formulations such as drug content uniformity are important for achieving consistent doses of drugs in the production batches, as this eliminates instances of overdosing or underdosing [31]. Therapeutic outcomes of formulations are suboptimal when the drug content deviates from the standard of 85%–115% WHO [50]. From the study carried out, none of the weights of all MOE-IR capsules deviated from the criteria stipulated by the British Pharmacopoeia [32, 47]. Furthermore, the British Pharmacopoeia states that to attain the required therapeutic effect, the disintegration time of a drug should not exceed 30 min. Disintegration influences the dissolution and absorption of a drug, which in turn determines drug bioavailability. Since disintegration time was within the acceptable range, it can be suggested that all the formulations would release the extract in requisite time for therapeutic action [30, 32]. Dissolution, which evaluates the rate and extent of how a drug gets into solution, is also essential for drug bioavailability and therapeutic effectiveness. Our study revealed that generally MOE-IR F_1_ (10 mg) showed a better dissolution profile in all media used than MOE-IR F_2_. Moreover, the MOE-IR F_2_ formulation also showed better characteristics than the crude extract. British Pharmacopoeia standards require that not less than 70% of the drug should be released in 45 min [47]. This requirement was met in the evaluation of MOE-IR F_1_ (10 mg) dosage form. It is worth noting that the dissolution rate decreased with increasing doses of *M. oppositifolius*, suggesting the creation of a supersaturated system with decreased dissolution. At low doses, less amount of the drug is exposed to the dissolution medium; hence, the solvent readily dissolves it, maintaining sink conditions. Conversely, at high doses, a larger mass of less soluble particles is introduced, which can aggregate into a thick concentrated layer at the solid–liquid interface, which can serve as a barrier preventing the fresh solvent from reaching its core, hindering its dissolution [51]. This is true for hydrophobic drugs like *M. oppositifolius*. Although this phenomenon is not uncommon with dissolution studies, the situation could be ameliorated with the inclusion of super disintegrants in the formulation. Taken together, MOE-IR F_1_ had the best properties followed by MOE-IR F_2_ and then the crude extract. Thus, the MOE-IR F_1_, subsequently referred to as MOE-IR, was selected for further proof-of-concept antidepressant studies.

In assessing the antidepressant-like effect of MOE-IR, the UCMS in tandem with the SPT was used. Anhedonia, which is demonstrated as decreased preference for sucrose in stressed mice, is a primary index for assessing depressive behavior [52]. Depressed animals demonstrate decreased preference for sucrose, while putative antidepressants reverse this. Although the chronic stress induced a consistent decline in sucrose preference, treatment with MOE-IR and FLX reversed this stress-induced reduction in sucrose preference. This is suggestive of the antidepressant-like effect. Interestingly, sucrose preference was restored to levels comparable to the nonstressed state by MOE-IR formulation. The antidepressant-like effect of MOE-IR was sustained 24 h after treatment was discontinued, suggestive of a sustained antidepressant-like effect. This was evidenced by the diminution in immobility scores in the FST and TST. The results corroborate previous findings where MOE showed a sustained antidepressant-like effect in mice [14]. Since the MOE-IR effect was associated with increased swimming and swinging scores, we hypothesize that increased serotoninergic and noradrenergic neurotransmission may contribute to the observed antidepressant effect. Serotonin and noradrenaline are important neurotransmitters that regulate mood, and their increased activity is relevant in the reversal of depressive symptoms. This finding lends support to previous studies by Kukuia et al. [15] who reported that the hydroethanolic extract of MOE mediates its antidepressant effect via the enhancement of serotonin and noradrenergic pathways. In that study, it was suggested that drugs that increase swimming score enhance serotoninergic neurotransmission, while those that increase swinging score increase both serotoninergic and noradrenergic neurotransmission. Moreover, a recent study showed that MOE induced an increase in serotonin levels in the PFC in mice [29]. This brain area is implicated in depression and an increase in serotonin levels may mediate the increase swimming and swinging scores. Some psychostimulants produce false positives in antidepressant research since they could reduce immobility scores without necessarily being antidepressants [45]. The open field test is thus used to rule out the influence of psychostimulant activity on the antidepressant-like effect observed. It is worth noting that the antidepressant-like effect of MOE-IR was not influenced by increased psychostimulant activity since the formulation did not significantly affect locomotor activity in the OFT [41, 44, 45]. Weight loss is a common phenomenon in clinical depression and in the UCMS [2, 53, 54]. A previous study suggested that the crude extract was not associated with a decrease in weight in depressed mice [14]; thus, the study investigated whether the MOE-IR would demonstrate results consistent with the previous one. In this current study, the antidepressant-like effect of MOE-IR was associated with a reversal in stress-induced weight just as reported in the previous study.

Recent reports have highlighted the role of hippocampal oxidative stress in the development of depressive-like behavior in rodents [55, 56]. Although moderate levels of reactive oxygen species (ROS) are needed for neuronal development, excessive levels are implicated in neuro-inflammation and its associated increase in cytokines decreased neurotrophic factors and neurogenesis as well as neurodegeneration [57]. Recent studies have suggested that depression is associated with low antioxidant capacity and reduced levels of plasma antioxidants such as Vitamin C, Vitamin E, and Coenzyme Q [58]. The reduced antioxidant capacity coupled with increased ROS implies the brain cells are unprotected against the damaging effects of the excess ROS. These mechanisms underlie depression neurobiology. Moreover, it has been shown that antioxidants have clinical benefits in the management of depression [59]. Based on these findings, in vitro antioxidant activity of MOE-IR was evaluated using the 2,2-diphenyl-1-picryl-hydrazyl-hydrate radical assay. Consistent with the study by Nwaehujor et al. (2014), both MOE crude and MOE-IR showed an antioxidant activity, although MOE-IR performed better. The enhanced antioxidant activity of the crude extract at 10 μg/mL compared to the formulation likely reflects greater bioaccessibility of antioxidant compounds at low concentrations, promoting efficient solubilization and immediate free radical scavenging. The formulated product at 10 μg/mL, though designed to go into solution quickly, may not be instantaneous at such low concentrations since the excipients may not fully dissolve instantly trapping a portion of the active compounds or slowing their release just enough that the initial reaction rate is slower than that of the predissolved crude extract. We hypothesize that the antidepressant-like effect of MOE-IR is partly due to its antioxidant activity. Generally, our study presents an IR formulation of *M. oppositifolius* that has an antidepressant-like effect, attenuated depression-related weight loss, and demonstrated antioxidant activity.

To understand the mechanism underlying the antidepressant-like effect of MOE-IR, the plasma levels of corticosterone were measured using ELISA. Dysfunction of the hypothalamus–pituitary–adrenal (HPA) axis in response to stress causes increased circulating corticosterone or cortisol and associated increased brain glucocorticoid receptors, which may lead to apoptosis and atrophy in the hippocampus and PFC [60]. Thakare et al. [61] have documented the correlation between elevated corticosteroids and behavioral alterations observed in depressed patients including fear, anxiety, and other psychological alterations [61]. Also, both clinical and preclinical studies confirm that depressed patients or animals have higher cortisol or corticosterone levels when compared to nondepressed subjects. Consequently, antidepressants have been shown to reduce the levels of these glucocorticoids [60]. In this study, we observed that MOE-IR and FLX reversed the stress-related elevation in serum corticosterone. This suggests that the antidepressant-like effect of MOE-IR is partly mediated through the inhibition of the HPA axis dysregulation. This assertion requires further investigation.

Another study carried out to elucidate the mode of action of MOE-IR was the evaluation of dendritic spines in the DG of the hippocampus. Previous studies by Ambe et al. [62] demonstrated that the hydro-ethanolic extract of *M. oppositifolius* showed no cytotoxicity but rather mitogenicity via the stimulated proliferation of progenitor neural cells (PNCs). A study by Kukuia et al. [29] also demonstrated that MOE produced an increase in the PFC dendritic spine density in depression-associated aggressive mice. In this study, we evaluated the effect of MOE-IR on the hippocampal neurons, specifically the DG . The hippocampus plays a crucial role in depression neurobiology. Various areas in the hippocampus (CA1, CA3, and DG) have been shown to be altered in stressed-induced depression [63]. Chronic stress inhibits neurogenesis in the DG while antidepressants reverse it [63]. Moreover, both preclinical and clinical studies suggest that stress-induced depression is associated with reduction in hippocampal volume. This reduction may be due to a decrease in neurogenesis, increased neuronal cell death, or changes in synaptic density [63, 64]. Considering the importance of the DG in processing information to the CA3 region of the hippocampus, it is no wonder that stress-caused changes in its neurons, in turn, influence depression symptomatology [64]. In our study, we focused on the granular layer neurons, the most abundant cell type in the DG . Our observations seem to suggest that stress had a negative impact on various apical dendritic spine types of the granular neurons. It is plausible that stress-induced decreased neurogenesis or increased neuronal cell death of the granular neurons contributed to our observation. In contrast, both MOE-IR and FLX increased the apical mushroom and stubby type dendritic spines. The mushroom-type spines are long-lived dendritic spines usually observed in mature neurons, which are involved in behavioral regulation [65, 66]. Their numerical reduction in stressed mice may explain the depressive behavior [67]. The MOE-IR-associated increase in mushroom spines may indicate that it protected mature granular layer neurons in the DG from death. Although the study did not investigate the actual mechanisms underlying this observation, we hypothesize that the MOE-IR may employ increased neurogenesis, decreased apoptosis, and/or increased dendritic spine density. The antioxidant activity and inhibitory effect on HPA axis dysregulation may contribute to its ability to prevent stressed-induced neuronal loss [68]. As previously reported, MOE increases serotoninergic neurotransmission [15, 29]. Serotonin concentration is implicated in the increased neurogenesis of the hippocampus. MOE's effect on serotonin contributed to the increased spine density in the DG [69].

In summary, our study has demonstrated the relevance of formulating crude extracts into an appropriate dosage form. *M. oppositifolius* leaf extract formulations exhibited a significant antioxidant effect over the crude extract. We also showed that the formulation retained the antidepressant-like capacity of the crude extract previously reported [15] and prevented depression-associated dendritic spines loss.

### 4.1. Limitations

Despite the important findings unearthed, our study was limited by our inability to explain the mechanism underlying the increased spine density. Future directions for this study include investigating the roles of glycine/NMDA receptor and BDNF/Trk B signaling pathways in the antidepressant-like effect of MOE-IR. Also, only male mice were used in this study. Considering the influence of sex differences on depression studies, future studies could consider investigating the comparative effect of the extract on female and male mice. Also, in vivo pharmacokinetic analysis would be ideal to predict the behavior of the drug in the body, which is currently out of scope of this work. Future studies on the formulation will focus on isolating and purify compounds responsible for antidepressant activity whose kinetics can be measured in vivo. Finally, a sustained release formulation could be tested to determine whether the previous faster antidepressant effect of the crude extract would be replicated.

## 5. Conclusion

The study has demonstrated that MOE-IR has an antidepressant-like effect in mice that is associated with antioxidant activity, reversal in weight loss, reduction in corticosterone levels, and increased DG dendritic spine density.

## Figures and Tables

**Table 1:** Proportions of MOE-IR and excipients for immediate-release formulation.

SN	*Mallotus* (mg)	Microcrystalline cellulose (mg)	Carboxymethyl cellulose (mg)	Lactose (mg)	Water	Total weight (mg)
MOE-IR F_1_	10	35	17.5	287.5	Qs	350
MOE-IR F_2_	10	35	10.5	277.0	Qs	350
MOE-IR F_1_	30	35	17.5	267.5	Qs	350
MOE-IR F_2_	30	35	10.5	274.5	Qs	350
MOE-IR F_1_	100	35	17.5	197.5	Qs	350
MOE-IR F_2_	100	35	10.5	187.0	Qs	350

Abbreviation: Qs, quantity sufficient.

**Table 2:** Drug content of immediate-release formulations of *M. oppositifolius* extract.

Formulation	Drug content (%)
MOE-IR F_1_ (10 mg)	105.27 ± 2.28
MOE-IR F_1_ (30 mg)	103.59 ± 2.67
MOE-IR F_1_ (100 mg)	105.27 ± 1.27
MOE-IR F_2_ (10 mg)	91.46 ± 4.55
MOE-IR F_2_ (30 mg)	102.91 ± 3.92
MOE-IR F_2_ (100 mg)	94.16 ± 1.270

**Table 3:** Powder flowability properties of *M. oppositifolius* extract.

Formulation	Angle of repose	Hausner ratio	Carr's index
MOE-IR F_1_ (10 mg)	24.9 ± 0.22	1.04 ± 0.03	4.0 ± 0.04
MOE-IR F_1_ (30 mg)	32.7 ± 0.06	1.12 ± 0.02	10.53 ± 0.03
MOE-IR F_1_ (100 mg)	35.6 ± 0.13	1.20 ± 0.01	16.95 ± 0.04
MOE-IR F_2_ (10 mg)	26.4 ± 0.04	1.05 ± 0.02	4.84 ± 0.02
MOE-IR F_2_ (30 mg)	32.8 ± 0.08	1.18 ± 0.02	15.09 ± 0.01
MOE-IR F_2_ (100 mg)	40.3 ± 0.02	1.35 ± 0.01	26.03 ± 0.09

**Table 4:** Disintegration times of immediate-release formulations of *M. oppositifolius* extract.

Formulation	Disintegration time (min)
MOE-IR F_1_ (10 mg)	8.33 ± 0.023
MOE-IR F_1_ (30 mg)	8.50 ± 0.017
MOE-IR F_1_ (100 mg)	8.58 ± 0.012
MOE-IR F_2_ (10 mg)	8.40 ± 0.020
MOE-IR F_2_ (30 mg)	8.45 ± 0.016
MOE-IR F_2_ (100 mg)	8.48 ± 0.018

**Table 5:** Characteristics of dendritic spines in the hippocampus of mice.

Spine characteristics	Treatment mg/kg (*n* = sample size)					
	VEH-NS (*n* = 40)	VEH-S (*n* = 24)	MOE-IR 10 (*n* = 35)	MOE-IR 30 (*n* = 38)	MOE-IR 100 (*n* = 32)	FLX (*n* = 38)
Mushroom (width > 0.6 µm)	1 (2.5%)	2 (8.3%)	5 (14.3%)	5 (15.6%)	3 (8.3%)	5 (13.2%)
Filopodia (length > 2 µm)	1 (2.5%)	1 (4.2%)	1 (2.9%)	2 (3.3%)	0 (0.0%)	1 (2.6%)
Stubby (LWR < 1 µm)	1 (2.5%)	3 (12.5%)	2 (5.7%)	0 (0.0%)	4 (12.5%)	8 (21.0%)
Long thin (length < 1 µm)	4 (10.0%)	2 (8.3%)	5 (14.3%)	3 (5.3%)	3 (9.4%)	4 (10.5%)
Thin (length < 2 µm)	28 (57.5%)	13 (54.2%)	14 (40.0%)	22 (57.9%)	14 (43.8%)	16 (42.1%)
Branch	10 (25.0%)	3 (12.5%)	8 (22.9%)	40 (10.5%)	6 (18.8%)	5 (13.2%)

*Note: n* represents the number of dendritic spines. The figures in brackets represent the percentage composition of each spine type in relation to the dendrites analyzed.

Abbreviation: LWR, length-to-width ratio.

## Data Availability

The data supporting this article will be made available by the authors, without undue reservation.

## Nomenclature

5-HT: 5-hydroxytriptamine
BP: British Pharmacopoeia
CNS: Central nervous system
FLX: Fluoxetine
FST: Forced swim test
IR: Immediate release
MOE: *Mallotus oppositifolius* extract
NMDA: N-methyl-D-aspartate
SSRI: Selective serotonin reuptake inhibitor
TCA: Tricyclic antidepressant
TST: Tail suspension test
UCMS: Unpredictable chronic mild stress
USP: U.S. Pharmacopoeia
